# Editorial: Traditional and up-to-date genomic insights into domestic animal diversity

**DOI:** 10.3389/fgene.2022.1117708

**Published:** 2023-01-04

**Authors:** Michael N. Romanov, Johann Sölkner, Natalia A. Zinovieva, Klaus Wimmers, Steffen Weigend

**Affiliations:** ^1^ School of Biosciences, University of Kent, Canterbury, United Kingdom; ^2^ Institute of Livestock Sciences (NUWI), University of Natural Resources and Life Sciences Vienna, Vienna, Austria; ^3^ L.K. Ernst Federal Science Center for Animal Husbandry (RAS), Moscow, Russia; ^4^ Research Institute for Farm Animal Biology (FBN), Dummerstorf, Germany; ^5^ Institute of Farm Animal Genetics, Friedrich-Loeffler-Institut (FLI), Neustadt, Germany

**Keywords:** domestic animal diversity, phenotypic traits, genomics, SNP genotyping, transcriptome, candidate genes, selective sweep, disease resistance

Domesticated animals play a significant role in local, national, and international agricultural output as well as in daily human life and culture. Additionally, they make up a sizeable portion of the biodiversity of the planet, which is essential for producing food and other animal products for human consumption. The present *Frontiers in Genetics* Research Topic (Figure 1) is devoted to various issues pertinent diversity of farm animals. The latter is at serious risk today, which could result in a reduction in the resources available to produce breed-specific food products and other necessities of everyday living. Importantly, genetic diversity is necessary for future animal breeding to be flexible enough to adapt livestock populations to changing customer demands and climatic conditions. Continued efforts are required to protect biodiversity, stop the loss of animal breeds, and maintain genetic diversity and develop strategies to use resource population in regional (niche) production systems.

**FIGURE 1:**
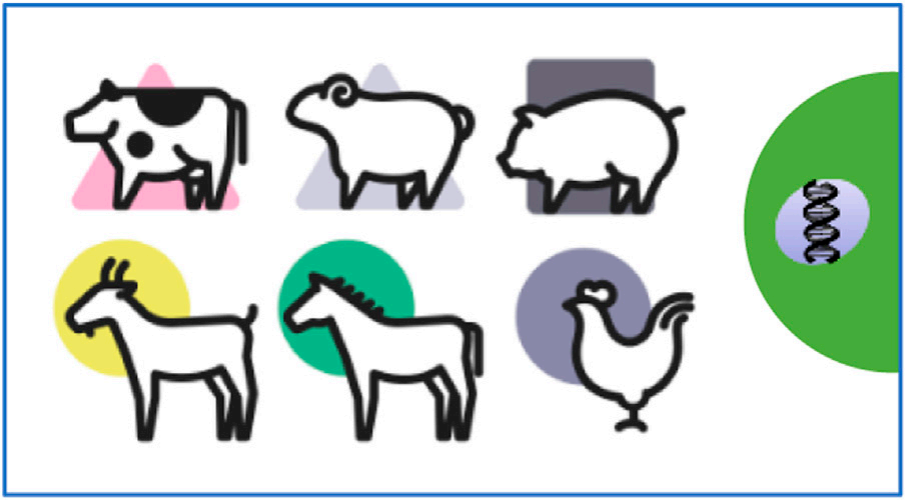
An artistic “logotype” for the *Frontiers in Genetics* Research Topic “Traditional and Up-to-date Genomic Insights into Domestic Animal Diversity”.

One of conventional ways to preserve the domestic animal diversity is the use of semen cryo-conserved in gene banks that also opens plentiful opportunities for genomics studies as reviewed by Oldenbroek and Windig. Gene banks were established not long after the beginning of implementation of cryo-conserved semen in the principal farm animal species. The fundamental goal was, and still is, preserving the genetic variation of agricultural animals for future use. DNA data from animals in living populations as well as from sires that have been cryopreserved is now accessible. Combining their DNA data opens up three potential avenues: 1) expanding the gene bank’s Research Topic of genetic diversity; 2) tracing the evolution of the genetic diversity from living populations; and 3) enhancing the genetic diversity and performance of existing populations. These three possibilities for the use of gene bank sires in the genomic era have been detailed in many studies demonstrating the immense significance of a gene bank as a library of genetic variation.

For conservation and preservation measures, the identification and evaluation of important genetic resources is necessary. This requires a comprehensive characterization of populations at risk of loss. This includes recording the phenotypes and especially those traits for which superiority and potential usefulness of the populations is anticipated. Characterization also includes the determination of genetic variability and distance. Genetic markers are used for this purpose. Advances in genome sequencing, the availability of genotyping tools and techniques such as SNP microarrays and genotyping-by-sequencing make such analyses simple, fast and informative. Ongoing progress in genome annotation increasingly enables the assessment of the functional significance of polymorphisms. The use of holistic “omics” techniques, especially next-generation sequencing, also enables a comprehensive characterization of genetic resources along the genotype–phenotype map at the genome, epigenome, transcriptome, proteome and metabolome levels. Such analyses not only contribute to the comprehensive characterization of genetic resources, but also allow the use of selected populations with specific traits, e.g., adaptation to environmental and climatic stresses or pathogens, to facilitate the understanding of the expression of functional traits and adaptation mechanisms to abiotic and biotic stressors, as shown in several studies in this Research Topic. Old, local, site-adapted breeds can thus serve as valuable models. A number of good research publications as overviewed below by species have been provided within the present Research Topic that address conventional methodologies, in addition to genotypic and genomic data, and more recent developments in the study and conservation of domestic animal diversity.

## Cattle

Kunene et al. used high-density single nucleotide polymorphism (SNP) genotypes to explore the genetics of base coat color variations and coat color patterns in South African Nguni cattle. The Nguni breed of cattle, which is similar to the Sanga, has mixed *B. taurus* and *B. indicus* lineage and has been shown to be resistant to ticks, illnesses, and other challenging environmental factors found in Africa. The leather business has developed a specialty market for the multicolored Nguni coats, which has prompted breeding goals for the propagation of such diversity. Limited research has been done on the genetic architecture that underlies coat color and pattern, which is a barrier to future breeding and development of that characteristic. Using Illumina Bovine HD (770K) genotypes and coat color phenotyped Nguni cattle, the authors examined genes underlying the base coat color, color-sidedness, and white forehead stripe in this cattle. Four indicative SNPs were identified on BTA18 as a result of the genome-wide association studies (GWAS) for base coat color (eumelanin vs. pheomelanin). A well-known gene, *MC1R*, was found to be located within 1 MB of the indicative SNPs, and it was discovered to be involved in both the mitogen-activated protein kinase (MAPK) signaling pathway and melanogenesis, the core pathway for the production of melanin. Four suggestive SNPs were discovered by GWAS for the color-sidedness gene, although none of them were located near the KIT candidate gene, which is linked to color-sidedness. Seventeen suggestive SNPs were found on BTA6 as a result of GWAS for the white forehead stripe. Four genes, including *MAPK10*, *EFNA5*, *PPP2R3C*, and *PAK1*, were discovered to be connected to the white forehead stripe and to the MAPK, adrenergic, and Wnt signaling pathways, which are mutually connected to melanin formation. These findings supported past theories about the function of *MC1R* in base coat colors in cattle and proposed a separate genetic mechanism for the phenotypes of Nguni cattle with forehead stripes.

Jaafar et al. evaluated the effects of employing several ancestral reference populations on the population admixture and performance of crossbred cattle. Animals from several breeds are bred together during the process of crossbreeding. The offspring show a combination of genetic improvements from the parental breeds that boost heterozygosity and counteract inbreeding depression, both additive and non-additive. However, because the advantages of heterosis rely on the type of crossbreeding systems utilized and the heritability of the traits, crossbreeding may also disrupt the special and frequently advantageous gene combinations in parental breeds, thus lowering performance potential. Regarding three-breed crossbreeding systems, it is yet unclear how crossbreeding affects the genomic architecture in particular. In order to compare genomic ancestry estimates to pedigree-based estimates, this study connected breed composition with important production and health parameters among two rotational crossbred populations, ProCROSS and Grazecross. Rotational crossbreeding of the Viking Red (VKR; a marketing name for Swedish Red, Danish Red, and Finnish Ayrshire breeds), Holstein, and Montbeliarde led to the creation of ProCROSS. In contrast, Grazecross was made up of VKR, Normande, and Jersey. Both breeding initiatives attempted to maximize heterosis’ beneficial effects. All genomic estimations considered, choosing the most appropriate and useful animals to use as the reference animals in admixture analysis is important when interpreting relationship and population structure results, but there is some uncertainty when determining how breed composition relates to phenotypic performance.

Long non-coding RNAs (lncRNAs) were identified and described by Liu et al. on a genome-wide scale in the *longissimus dorsi* skeletal muscle of Shandong Black and Luxi cattle. LncRNAs may play a regulatory role, which is becoming increasingly clear. The regulation of cell differentiation, fat synthesis, and embryonic development have been the main Research Topic of studies on cattle. However, there has not been much research on the potential function of lncRNAs in the skeletal muscle of domestic animals. Here, bioinformatics analysis was employed to build a network of lncRNAs, miRNAs, and mRNA interactions connected to muscle using the transcriptome numbers of distinct beef cattle, Shandong Black and Luxi. This can be utilized to advance animal husbandry, increase animal husbandry output, and elucidate the molecular basis of the growth of bovine muscle. A total of 1,415 transcripts (of which 480 were lncRNAs) were differently expressed in the two breeds. Furthermore, 1,164 protein-coding genes (*MYORG*, *Wnt4*, *PAK1*, *ADCY7*, etc.) were the targets of the most differentially expressed lncRNAs, which were located on chromosome 9. A probable trans-regulatory link between the differentially expressed lncRNAs and 43,844 mRNAs was also shown. The detected co-expressed mRNAs (*MYORG*, *Dll1*, *EFNB2*, *SOX6*, *MYOCD*, and *MYLK3*) are enriched in calcium and AMPK signaling, muscle cell and striated muscle tissue development, and strained muscle cell differentiation. A network of lncRNAs, miRNAs, and mRNA interactions was built as the putative foundation for biological control in the skeletal muscle of cattle based on this. The reported findings will theoretically support future research on lncRNA regulation and activity in various cattle breeds.

## Yak

Bao et al. used resequencing to examine the signals of selective scanning leading to potential genetic variations for hair length features in Tianzhu white yaks with long and normal hair. The Tianzhu white yak is a unique native yak breed in China that has an all-white coat. Breeders have recently identified long-haired individuals of the Tianzhu white yak, which are distinguished by long hair on the forehead. The length and density of the hair on these two sections of the body are also higher than that of the typical Tianzhu white yak. The authors re-sequenced the whole genomes of long-haired and normal Tianzhu White yaks to clarify the genetic basis of hair length in Tianzhu white yaks. Two hotspots were discovered on chromosome 6 that contain two (*FGF5* and *CFAP299*) and four (*ATP8A1*, *SLC30A9*, *SHISA3*, and *TMEM33*) genes, respectively. Ras, MAPK, PI3K-Akt and Rap1 signaling pathways were found to be involved in the process of hair length variation by function enrichment analysis of genes in two hotspots. In addition, four more genes (*ACOXL*, *PDPK1*, *MAGEL2*, and *CDH1*) were discovered as connected to the growth of hair follicles.

## Pig

Using a whole-genome SNP chip, Yuan et al. investigated the genetic diversity and population dynamics of Tongcheng (TC) pigs. Indigenous to China, TC pigs are known for their high meat quality. Due to the introduction of global pig breeds and the African swine fever pathogen, the genetic resources of TC pigs are now seriously threatened. The current study used multiple SNP markers to analyze the genetic diversity and population structure of TC pigs in order to support their management and conservation. With an average linkage disequilibrium (LD) value of .15, LD and neutrality testing both showed a low selection of TC pigs. Estimates of minor allele frequency, observed heterozygosity (*H*_*o*_), expected heterozygosity, and nucleotide diversity values pointed to the TC pigs’ astonishingly great genetic diversity. Additionally, runs of homozygosity (ROHs) segments were examined in the whole genome of TC pigs. Based on ROHs, the average genomic inbreeding coefficient *F*_ROH_ was .04%. On nine separate autosomes, 14 ROH islands with 240 genes were discovered. Some of them, including *FFAR2*, *FFAR4*, *MAPK8*, *NPY5R*, and *KISS1*, overlapped with genes involved in immunological response, reproduction, muscle development, and fat deposition. These genes may be linked to qualities like meat quality and disease resistance in TC pigs. Genetic diversity and population structure data together revealed that the TC pig was a valuable genetic resource. To provide sufficient genetic diversity and prevent inbreeding depression, the TC pig breed conservation program needs to be further developed, and this research gives management and conservation methods for TC pigs a theoretical foundation.

Insights on the ROH distribution and breed differentiation in Mangalitsa pigs were provided by Addo and Jung. Mangalitsa pigs have three different color patterns on their coats, according to which they can be classified as Red, Blond, or Swallow-bellied. The current work used studies of population structure, ROHs, and fixation index to examine genome-wide diversity and selection fingerprints in the three breeds genotyped using a modified ProcineSNP60 v2 Genotyping Bead Chip. Also provided as comparison outgroup data were 20 genotypes of the Hungarian Mangalitsa. For the Blond, Swallow-bellied, and Red Mangalitsa, respectively, estimates of observed heterozygosity were .27, .28, and .29, and estimates of inbreeding coefficients based on ROHs were 24.11%, 20.82%, and 16.34%. All breeds had ROH islands, but none of them were shared by any of the breeds. In a ROH island in the Swallow-bellied Mangalitsa, the *KIF16B* gene—previously known to be involved in synaptic signaling—was discovered. The same gene was discovered to contain a significantly different SNP (MARC0032380) when comparing Swallow-belied Mangalitsa to either Red or Blond. Some ROH islands in the Red Mangalitsa were connected to genes like *ABCA12*, *VIL1*, *PLSCR5*, and *USP37* that affect meat quality attributes. This research revealed that the variation and population structure of the three breeds were distinct, with the Red and Blond Mangalitsa pigs being the most closely related.

In a Chinese pig breed, Feng et al. discovered a putative miRNA-mRNA regulation network and the important miRNAs in intramuscular and subcutaneous adipose. Intramuscular fat (IMF) is a key metric for assessing the quality of meat. Breeds with high IMF content frequently also have high subcutaneous fat, which negatively impacts pigs’ ability to produce meat. Important ramifications for pig breeding result from research into the processes of miRNAs involved in lipogenesis and lipid metabolism. Here, the patterns of lipogenesis in the Chinese breed of pig known as Laiwu were analyzed by creating two small RNA libraries from intramuscular and subcutaneous fat. Two types of adipose tissue were used to identify a total of 286 differentially expressed miRNAs (DEmiRNAs), comprising 193 known miRNA and 93 novel miRNA. Gene ontology (GO) and KEGG enrichment analysis for DEmiRNAs revealed that their target genes were involved in numerous biological processes and signaling pathways related to adipogenesis and lipid metabolism, including the Wnt, MAPK, Hippo and PI3K-Akt signaling pathways, melanogenesis, and signaling pathways controlling stem cell pluripotency, among others. After that, a network of interactions between miRNA and mRNA was built to determine which miRNAs were crucial for regulating the Wnt signaling pathway. MiR-331-3p, miR-339-5p, miR-874, and novel346 mature target *PPARD*, *WNT10B*, *RSPO3*, and *WNT2B* in this pathway. This research offers a theoretical foundation for future research into the post-transcriptional control mechanism of meat quality generation as well as disease diagnosis and management related to ectopic fat.

An integrative analysis of the alveolar type II epithelial (ATII) cells of Tibetan pigs and their response to hypoxia was conducted by Yang et al. with respect to the lncRNA-associated ceRNA regulation network. Understanding the regulatory mechanisms governing responses to hypoxia may help in alleviating harm brought on by hypoxia. ATII cells’ ability to function is significantly hindered by oxygen deprivation. In this study, ATII cells were cultivated from Tibetan and Landrace pigs in hypoxic and normoxic conditions to search for differentially expressed (DE) lncRNAs and DEmiRNAs as well as build their related ceRNA regulation networks in response to hypoxia. Target genes of Tibetan and Landrace pig DElncRNAs were significantly enriched in the proteoglycans in cancer, renal cell carcinoma, and erbB signaling pathways between the normoxic and hypoxic groups, whereas DEmiRNAs’ target genes were significantly enriched in the axon guidance, focal adhesion, and MAPK signaling pathways. Through the activation of the focal adhesion/PI3K-Akt/glycolysis pathway, hypoxia induction has been demonstrated to potentially encourage apoptosis. By controlling ATII cell autophagy in normoxic and hypoxic conditions, the ssc-miR-20b/MSTRG.57127.1/ssc-miR-7-5p axis may have significantly reduced hypoxia injury. The most impacted axis, MSTRG.14861.4-miR-11971-z-CCDC12, controlled a number of RNAs and may thus control the proliferation of ATII cells in Tibetan pigs under hypoxic settings. In Landrace pigs, the ACTA1/ssc-miR-30c-3p/MSTRG.23871.1 axis plays a critical role in reducing ATII cell damage and enhancing dysfunction and fibrosis brought on by oxidative stress. These findings give a better knowledge of how Tibetan pigs regulate their lncRNA, miRNA, and mRNA in hypoxic environments.

In Hulun Buir sheep, Li et al. discovered *SSTR5* gene polymorphisms and their correlation with growth traits. This investigation sought to identify *SSTR5* polymorphisms and assess their relationship to growth parameters in Hulun Buir sheep. Seven SNPs were found as a result of Sanger sequencing, showed moderate polymorphism (.25 < PIC <.5), and were then subject to association analysis in relation to the growth traits. At 9 months of age, cannon circumference was substantially related with SNP4 (rs605867745) and SNP3 (rs413380618). There was linkage disequilibrium among the five haplotypes and seven SNPs. These haplotypes were not connected to distinct ages of growth features, nevertheless. SNP1, SNP3, SNP4, and SNP7 may all function as molecular markers for the growth features of Hulun Buir sheep, to sum up.

A genome-wide divergence and selection signature investigation of South African Merino-derived breeds from their ancestors was carried out by Dzomba et al. Merino sheep are a preferred breed that are widely raised for their wool and mutton worth. Using the Illumina Ovine50K BeadChip, this study assessed genetic diversity, population structure, and breed divergence in the South African Merino (SAM), eight Merino-based sheep breeds, as well as non-Merino founding breeds (Damara, Ronderib Afrikaner, and Nguni). The Meatmaster, SAM and Dohne Merino (DM) showed the highest genetic diversity levels, with *H*_*o*_ values of .37–.39. The degree of inbreeding varied from zero (DM) to .27 points (Nguni). High within population variance (80 + per cent) was observed across all population categories. Selection sweeps for the Afrino (12 sweeps), Meatmaster (four sweeps), and DM (29 sweeps) were identified. Such genes as *FGF12*, the metabolic genes *ICA1*, *NXPH1*, and *GPR171*, as well as the immune response genes *IL22*, *IL26*, *IFNAR1*, and *IL10RB*, have all been linked to hair and wool features. The DM vs. Merino, Meatmaster vs. Merino and Meatmaster vs. Nguni shared a selection sweep on chromosome 10 harboring the *RXFP2* gene for the polledness. Additionally, the DM vs. Merino and the Meatmaster vs. Merino shared a Rsb-based selection sweep on chromosome 1 connected to the *CAPN7* gene for calpain. Collectively, the analysis showed some genetic divergence caused by breed-specific selection objectives and some genetic similarity between the Merino and Merino-derived breeds originating from shared founder populations.

High-density genomic characterization of native Croatian sheep breeds was performed by Drzaic et al. Using 50K SNP profiles, this comprehensive genomic research has revealed that the regional Balkan sheep populations share a great deal of genetic variation with neighboring breeds, but they are also very distinct from them. Using the Ovine Infinium^®^ HD SNP BeadChip, eight Croatian sheep breeds and mouflon were genotyped. Also, various Mediterranean sheep breeds and Balkan Pramenka, which are readily accessible, were added to the analysis. This research uncovered the intricate demographic structure of Croatian sheep breeds, as well as information on their geographic origins (island vs. mainland). The historical establishment of breeds and the routes of gene flow were confirmed by migration patterns. Between sheep populations, *F*_ROH>2 Mb_ coefficients ranged from .025 to .070, with Dalmatian Pramenka and Pag Island Sheep having lower inbreeding coefficients and Dubrovnik sheep having higher inbreeding. For the Krk Island Sheep and Dalmatian Pramenka, the estimated effective population size (*N*_*e*_) varied from 61 to 1039, respectively. In order to retain genetic variation in particular breeds, there is a need for greater conservation management due to higher inbreeding rates and a smaller *N*_*e*_. These findings will aid in breeding and developing conservation plans for Croatia’s indigenous sheep breeds.

A genome-wide investigation of miRNAs by Liu et al. revealed that the lipid metabolism pathway is a key characteristic of adipose tissue from various sheep. Important non-coding RNAs known as miRNAs can take part in the control of biological processes. MiRNAs have been extensively explored in recent years, not just in humans and mice but also in animal husbandry. However, the investigation of miRNA in subcutaneous adipose tissue of sheep is not thorough compared to other livestock and poultry breeds. Using RNA-Seq technology, the transcriptomes of miRNAs in the subcutaneous adipose tissue of Duolang sheep and Small Tail Han sheep were analyzed to identify those that were expressed differently in these two breeds. As a result, 38 miRNAs were discovered that were differently expressed (nine novel miRNAs and 29 known miRNAs). Additionally, 854 target genes were predicted in total. The deposition of subcutaneous adipose tissue in Duolang and Small Tail Han sheep has been linked to the regulation of lipolysis in adipocytes. The genes involved in controlling lipolysis in adipocytes may be controlled by the miRNAs, which in turn may control fat accumulation. In particular, NC_ 040278.1 37602, oar-mir-493-3p, NC_ 040278.1 37521, and NC_ 040255.1 11627 may each target *PTGS2*, *AKT2*, *AKT3*, and *PIK3CA*, thus playing a crucial regulatory role. Overall, the findings establish the groundwork for further elucidating the mechanism underlying the deposition of subcutaneous adipose tissue in sheep, enhancing the performance of their ability to produce meat, and advancing the field of animal husbandry.

## Goat

Using the genome-wide Illumina goat SNP50K BeadChip, Chokoe et al. clarified the conservation status and historical relatedness of communal indigenous goat populations in South Africa. Due to their tolerance to various production situations and support for communal farming, indigenous goats, which make up the bulk of populations in smallholder, low input, low output production systems, are regarded as an important genetic resource. In order to assist breeding strategies to utilize and conserve genetic resources, *N*_*e*_, inbreeding rates, and ROHs are useful tools for examining genetic diversity and comprehending the demographic history. The historical *N*_*e*_ across populations indicates that the ancestor *N*_*e*_ has decreased in Free State (FS), North West (NW), Limpopo (LP), and Gauteng (GP), respectively, over the last 971 generations. The current *N*_*e*_ of GP was the lowest across populations. The Eastern Cape (EC) had the lowest levels of *F*_ROH>5 Mb_, and FS had the greatest levels. The FS, GP and NW populations had 871 ROH island areas that contain crucial environmental adaptation and thermo-tolerance genes such *IL10RB*, *IL23A*, *FGF9*, *IGF1*, *EGR1*, and *MAPK3*. Despite having a similar ancestor, the genomes of KZN and LP exhibit significant mixing from the EC and NW populations. Using genome-wide SNP markers, the results showed that the presence of high *N*_*e*_ and autozygosity differed significantly across communal indigenous goat populations at recent and ancient events. The migration of communal indigenous goat populations from the northern section (LP) of South Africa to the eastern regions (KZN) showed their historical kinship and coincided with the Bantu nation’s migratory phases.

Translatomics can be used to select functional genes for cashmere fineness, as Zhang et al. demonstrated. An essential metric for assessing cashmere quality is cashmere fineness. Although the Liaoning Cashmere Goat (LCG) breed studied produces a lot of cashmere and has long cashmere fiber, its fineness may be better. Thus, it is crucial to identify genes related to cashmere fineness that might be applied in next attempts to enhance this phenotype. Through high-throughput sequencing and genome-wide association analysis, the regulation of cashmere fineness has achieved unprecedented strides due to the ongoing improvement of technology. Translatomics has been demonstrated to be able to pinpoint genes linked to phenotypic features. The authors performed translatomic analysis after having sequenced the skin tissue of LCG sample groups with various cashmere fineness ranges by Ribo-seq. Differently expressed genes were found between the sample groups using these data. From these, 186 genes were downregulated and 343 genes were upregulated in the fine LCG group as compared to the coarse LCG group. The biological functions of differential genes were explored by GO enrichment analysis, with functional genes related to the extracellular area being predominant. The enrichment of the human papillomavirus infection pathway was particularly prominent in the KEGG enrichment study. The authors suggested that the *COL6A5* gene might impact the fineness of cashmere.

## Poultry

In highly productive lines of laying hens, Iqbal et al. investigated how multi-omics can disclose different strategies in the immune and metabolic systems. Due to their high egg production and outstanding commercial applicability, Lohmann Brown (LB) and Lohmann Selected Leghorn (LSL) are two major laying hen strains for the poultry industry. To further understand how the genetic makeup of the two strains effects their biological pathways, the genotype-phenotype map of the current study incorporated multiple data sets. The harvested data sets in the two strains were intestinal miRNA and mRNA transcriptome data, immune cells, inositol phosphate metabolites, minerals, and hormones from various organs. These were analyzed using the R package mixOmics. Among the most distinctive characteristics between the two strains, there were 20 miRNAs, 20 mRNAs, 16 immune cells, 10 microorganisms, 11 behavioral properties, and 16 metabolites. The enrichment of immune pathways in the LSL strain was correlated with the expression of particular miRNAs and the quantity of different immune cell types. On the other hand, more microbial taxa that were unique to the LB strain were discovered, and the prevalence of several bacteria showed a high correlation with transcripts enriched in immunological and metabolic pathways in the host gut. According to this research, both strains use unique innate mechanisms to develop and keep their immune and metabolic systems functioning well. The study also adds to our understanding of the role of host–gut interactions, including immune phenotype, microbiota, gut transcriptome, and metabolome, by offering a fresh perspective on the functional biodiversity that emerges during strain selection.

The utility of next-generation sequencing (NGS) for the high-resolution typing of major histocompatibility complex-B (MHC-B) in Korean native chickens (KNC) was proven by Ediriweera et al. The chicken MHC-B region plays a vital role in the development of the immune systems and is extremely variable across breeds, lines, and populations. It is crucial to examine this chromosomal region, particularly the class I and II genes, to ascertain the variation and diversity that eventually alter antigen presentation because it determines the resistance/susceptibility to a variety of infectious illnesses. Using NGS data, Geneious Prime-based assembly and variant calling with the Genome Analysis Toolkit (GATK) best practices pipeline, this study examined five KNC lines and the Ogye breed. For each line or breed of chicken, the consensus MHC-B (*BG1*–*BF2*) sequences were collected, and their variations were examined. Each of the KNC lines had an excessive number of mutations, including a sizable number of high-impact variants that revealed important details about altered MHC molecules. The study verified that MHC variations can be successfully detected using NGS techniques, and the KNC lines had a very diverse MHC-B region, indicating a significant divergence from the red junglefowl, the progenitor of domestic fowls.

Time-series transcriptome analysis revealed differently expressed genes in broiler chicken infected with mixed *Eimeria* species, as demonstrated by Kim et al. The coccidiosis disease brought on by the *Eimeria* species is quite harmful for the poultry production. RNA sequencing was employed to track the temporally-dependent host responses of chickens infected with *Eimeria* in order to investigate the genes and biological processes linked to parasite immunity. Four, seven, and 21 days post infection (dpi) were the times at which transcriptome analysis was carried out. Three categories of genes with differential expression were identified based on alterations in gene expression patterns. As a result, endoplasmic reticulum stress was document during the early stages of *Eimeria* infection. Furthermore, innate immune responses to the parasite were engaged at the time of the initial exposure and gradually returned to normal. Despite being considerably active at 4 dpi, the cytokine-cytokine receptor interaction pathway was downregulated, which had an anti-inflammatory effect. After *Eimeria* infection, the creation of gene co-expression networks also made it possible to identify key pattern recognition receptors and immunoregulation hub genes. These findings give a thorough insight of how chickens and *Eimeria* interact as hosts and pathogens. The gene clusters identified in this work can be used to enhance the coccidiosis resistance of chickens.

In conclusion, the evaluation of breeds by comprehensive phenotyping and the effective study of breed genetic diversity using appropriate polymorphic markers or genome-wide SNP genotyping are both possible today thanks to the combination of traditional techniques and approaches with cutting-edge genetic and genomic methods. In this way, the genotype/genomic content is important and gains from the comparison or combination of conventional and genomic metrics. The papers in this Research Topic serve as excellent illustrations for how to create and contrast “portraits” of breeds at the level of complete genomic sequences and transcriptomes, as well as how to identify potential candidate genes for key features in particular breeds. The history of domestication and the development of breeds can be clarified using genomic data, and genomic regions with signs of artificial selection can be found. The formulation of breed conservation measures, their sustainable use, and marker-assisted breeding are all made possible with the help of comprehensive breed assessment using genetic, genomic and multi-omic approaches.

